# Concentration of Donepezil in the Cerebrospinal Fluid of AD Patients: Evaluation of Dosage Sufficiency in Standard Treatment Strategy

**DOI:** 10.1007/s12640-016-9672-y

**Published:** 2016-10-07

**Authors:** Martin Valis, Jiri Masopust, Oldrich Vysata, Jakub Hort, Rafael Dolezal, Jiri Tomek, Jan Misik, Kamil Kuca, Jana Zdarova Karasova

**Affiliations:** 1 Department of Neurology, Faculty of Medicine and University Hospital Hradec Kralove, Charles University in Prague, Hradec Kralove, Czech Republic; 2 Department of Psychiatry, Faculty of Medicine and University Hospital Hradec Kralove, Charles University in Prague, Hradec Kralove, Czech Republic; 3 Department of Neurology, 2nd Faculty of Medicine, Charles University in Prague and Motol University Hospital, Prague, Czech Republic; 4 International Clinical Research Center, St. Anne’s University Hospital Brno, Brno, Czech Republic; 5 Biomedical Research Center, University Hospital Hradec Kralove, Hradec Kralove, Czech Republic; 6 Department of Toxicology and Military Pharmacy, Faculty of Military Health Sciences, University of Defense in Brno, Trebesska 1575, 500 01 Hradec Kralove, Czech Republic

**Keywords:** Alzheimer’s disease, Donepezil, Clinical study, Cerebrospinal fluid concentrations

## Abstract

Although some studies have described the pharmacokinetics and pharmacodynamics of donepezil in the peripheral compartment, studies focused on drug transport across the blood–brain barrier are still very rare. To our knowledge, the fluctuation in the cerebrospinal fluid concentration of donepezil after administration of the drug has not been described in the literature so far. We recruited 16 patients regularly taking a standard therapeutic dose of donepezil (10 mg per day). All patients (Caucasian race) were treated for at least three months with a stable dose of 10 mg per day prior to sample collection. Patients were divided into two groups depending on the time of plasma and cerebrospinal fluid sampling: 12 h (*n* = 9; 4 M/5F aged 78.68 ± 7.35 years) and 24 h (*n* = 7; 3 M/4F aged 77.14 ± 5.87 years) after donepezil administration. The cerebrospinal fluid sample was collected by standard lumbar puncture technique using a single-use traumatic needle. The samples were analysed on an Agilent 1260 Series liquid chromatograph comprising a degasser, a quaternary pump, a light-tight autosampler unit set, a thermostated column compartment, and a UV/VIS detector. Agilent ChemStation software, the statistical software Prism4, version 5.0 (GraphPad Software, USA), and IBM^®^ SPSS^®^ Statistics were used for the analysis of the results. The difference in plasma concentration of donepezil after 12 h (mean ± SEM; 39.99 ± 5.90 ng/ml) and after 24 h (29.38 ± 1.71 ng/ml) was nonsignificant. In contrast, the donepezil concentration in the cerebrospinal fluid was significantly higher in the 24-h interval (7.54 ± 0.55 ng/ml) compared with the 12-h interval (5.19 ± 0.83 ng/ml, which is ~70 % based on mean cerebrospinal fluid values). Based on these data, it is plausible to predict that donepezil might produce a stronger AChE inhibition in the brain at 24 h compared with 12 h following the administration. This information may help physicians individually adjust the time of drug administration in the patients according to time course of the disease symptoms.

## Introduction

Alzheimer’s disease (AD) is a progressive neurodegenerative disorder affecting a significant proportion of the elderly population. With respect to continual increase of life expectancy, AD is expected to affect more than 100 million individuals worldwide in the near future (Giacobini 2008). The aetiology of AD includes upstream and downstream processes in which cholinergic deficits are clinically relevant. Cholinergic deficits have been shown to be caused by degeneration of the subcortical regions, especially the hippocampal projections to the basal forebrain. Hippocampal cholinergic deficits are responsible for impaired memory processing (Talesa 2001). Inhibition of the enzyme acetylcholinesterase (AChE), which breaks down acetylcholine at the synapse, may be one method of increasing the concentration of this neurotransmitter in certain areas of the brain and may affect the symptoms of the disease. Some evidence suggests that the administration of rivastigmine, donepezil, or galantamine may be beneficial in patients with AD (Ritchie et al. 2004). The selective AChE inhibitor donepezil is widely used as a symptomatic treatment for the disease (Darreh-Shori et al. 2006). Donepezil is well absorbed, with a bioavailability approaching 100 %. Peak plasma concentrations (Cmax) are achieved after 3–5 h in patients receiving immediate-release (5- and 10-mg tablets) and 6 h in those receiving sustained-release (23-mg tablet) formulations (see Table 1; taken from Noetzli and Eap 2013). In the USA, the FDA-approved dose is higher at 23 mg daily. Although some studies have described the pharmacokinetics and pharmacodynamics of the drug in the peripheral compartment, studies focused on drug penetration across the blood–brain barrier are still very rare. To our knowledge, the changes in cerebrospinal fluid (CSF) concentrations between doses of donepezil have not been described in the literature to date.

**Table 1:** Steady-state pharmacokinetic parameters of donepezil (Noetzli and Eap 2013)

Parameter	Donepezil
Daily dose (mg)	5–10, 23^a^
Bioavailability (%)	100
Protein binding (%)	93
t_1/2_ (h)	70^b^
t_max,ss_ (h)	4 (IR), 6 (SR)
C_max,ss_ (ng/mL)	61 ± 10 (10 mg/day), 129 (CV 29 %) (23 mg/day)^a^
AUC_max,ss_ (ng h/mL)	1128 ± 196 (10 mg/day)^c^
CL_tot_ (L/h)	10 ± 2.5^c^
V_d_ (L/kg)	11 ± 2^c^
Metabolism	Hepatic (CYP2D6, CYP3A4, UGT)
Kinetics	Linear
Steady state (days)	14–21

*AUC*_*ss*_ area under the concentration–time curve at steady state, *cap* capsule; *CL*_*tot*_ total systemic clearance, *C*_*max,ss*_ maximum steady-state plasma drug concentration during a dosage interval, *CV* coefficient of variation, *CYP* cytochrome P450, *ER* extended-release formulation, *IR* immediate-release formulation, *SR* sustained-release formulation, *t*_*½*_ elimination half-life, *t*_*max,ss*_ time to reach C_*max,ss*_ *UGT* uridine 50-diphosphoglucuronosyltransferase, *V*_*d*_ apparent volume of distribution

^a^The new dosage of 23 mg has recently been approved by the US FDA for the treatment of moderate-to-severe Alzheimer’s disease

^b^The t_½_ of the drug is short, but the duration of action is longer because acetylcholinesterase and butyrylcholinesterase are inhibited for 8.5 and 3.5 h, respectively, through a pseudoirreversible mechanism

^c^Mean ± standard deviation or range

## Materials and Methods

### Sampling and Patients

Sixteen patients (Caucasian race) who regularly use a standard therapeutic dose of donepezil (10 mg per day) were recruited into the study. All patients were treated for at least 3 months with a stable dose of 10 mg per day prior to sample collection. The patients were randomly divided into two groups depending on the time of plasma and CSF sampling: 12 h (*n* = 9; 4 M/5F aged 78.68 ± 7.35 years) and 24 h (*n* = 7; 3 M/4F aged 77.14 ± 5.87 years) following donepezil administration.

All patients signed an informed consent to lumbar puncture, and the study was approved and supervised by the Local Ethics Committee. The study was progressed according to national and international guidelines (Ressner et al. 2008; Hort et al. 2010); the lumbar puncture was part of the standard diagnostic investigation (CSF tau and Aβ42 can be used as markers in patients suspected of having AD, but in whom the clinical diagnosis is uncertain). The cerebrospinal fluid sample was collected by a standard lumbar puncture technique using a single-use traumatic needle for the purpose of this research project. The diagnosis of probable AD was made by the exclusion of other types of dementia in accordance with the NINCDS-ADRDA criteria (National Institute of Neurological and Communication Disorders and Stroke-Alzheimer’s Disease and Related Disorders Association) (McKhann et al. 1984; Hort et al. 2010). Dementia was established by clinical and neuropsychological examination. Cognitive impairment also had to be progressive and presented in two or more areas of cognition. The onset of the deficit had to be recognised between 40 and 90 years of age, and finally other diseases capable of demonstrating as dementia syndrome had to be excluded.

### Chemicals

Chemicals for sample preparation and high-performance liquid chromatography (HPLC) measurement were purchased from Sigma-Aldrich (Prague branch, Czech Republic) and were of the best available quality: acetonitrile, gradient grade; LiChrosolv in analytical grade; n-hexane and 1-butanol in gradient grade. All other reagents used in analytical methods and sample preparation were of analytical grade. The water used in the study was double-distilled and was of deionised HPLC grade.

### Instrumentation

The samples were analysed on an Agilent 1260 Series liquid chromatograph (Palo Alto, CA, USA) composed of a degasser, a quaternary pump, a light-tight autosampler unit set, a thermostated column compartment, and a UV/VIS detector. Agilent ChemStation software (Palo Alto, CA, USA) and the statistical software Prism4 (GraphPad Software, USA) were used for data analysis.

### Separation Conditions for Donepezil in Plasma and CSF Samples

An analytical column (Waters Spherisorb S5 W/250 mm × 4.6 i.d.; 5 μm particle size/with guard column (Waters Spherisorb S5 W/30 mm × 4.6 mm i.d./) was used for the analysis. The mobile phase contained acetonitrile and 50 mM sodium dihydrogen phosphate (17:83; v/v). The pH was adjusted to 3.1 with phosphoric acid (Amini and Ahmadiani 2010). The sample volume was 40 µl. All chromatograms were obtained at a conditioned temperature (40 °C), and the flow rate was 1.3 ml/min. Rivastigmine was used as an internal standard (Amini and Ahmadiani 2010). The described analytical method is sensitive for donepezil and also for rivastigmine, and may be used for both acetylcholinesterase inhibitors. The samples were analysed by a UV detector, and the maximum wavelength of donepezil was 210 nm.

### Sample Preparation

Extraction was performed with 20 µl of 1 M sodium hydroxide and 3 ml of 1-butanol/n-hexane (2:98; v/v) to 1 ml plasma or CSF; subsequently, the samples were intensely shaken for 5 min and centrifuged at 11,000×*g* and 10 °C for 10 min. The entire organic layer was separated into a new tube, and re-extraction was performed with 100 µl of 0.1 % acetic acid (Amini and Ahmadiani 2010). The mixture was intensely shaken and centrifuged. The lower aqueous phase was separated and directly injected into the HPLC system.

### Preparation of Calibration Standards and Calculation of Assayed Concentrations

**Fig. 1:**
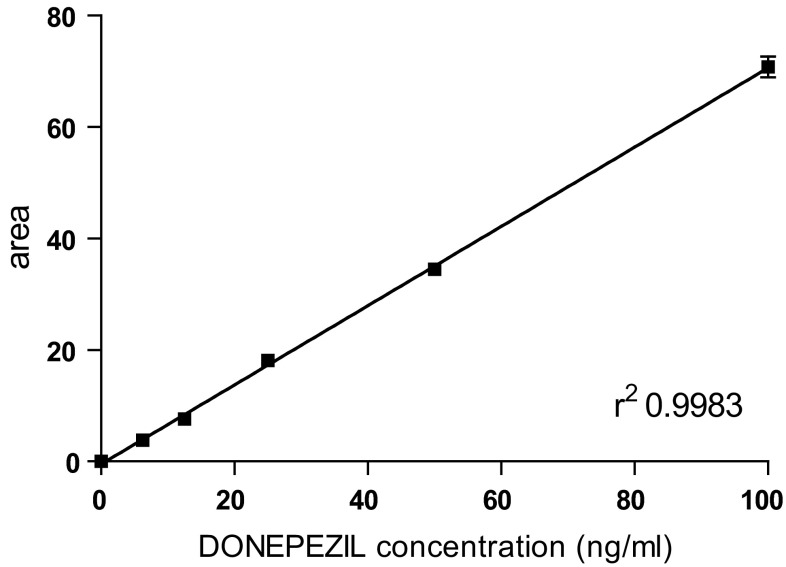
Calibration curve of donepezil in human CSF

Calibration standards were established using blank human plasma or human CSF supplemented with donepezil in a range of concentrations (0, 3.125, 6.25, 12.5, 25, 50, and 100 ng/ml; in triplicate); see Fig. 1.

Regression analysis of the relationship between peak areas and theoretical concentrations of calibration standards was performed with the least-squares method using Prism4 (GraphPad Software, USA). Endogenous compounds in the plasma and CSF were efficiently separated from donepezil by sample preparation and elution for 23 min. The donepezil concentration exhibited a linear relationship (*r* ^2^ > 0.9967 in plasma; *r* ^2^ > 0.9983 in CSF) between the peak areas and calibration standards in the range of 3.125–100 ng/ml in the plasma and CSF.

### Precision in Plasma and CSF Samples

Intra-day precision was determined by repeating the measurement of spiked samples ten times with five different concentrations of plasma and CSF (6.25, 12.5, 25, 50, and 100 ng/ml; *n* = 4) on the same day. Inter-day precision was determined by calculating the means of four replicates of samples spiked with donepezil (plasma and CSF: 6.25, 12.5, 25, 50, and 100 ng/ml) on 3 subsequent days.

The intra-day and inter-day coefficients of variations (C.V.) of donepezil are summarised in Table 2.

**Table 2:** Inter- and intra-day precision in plasma and cerebrospinal fluid samples

Plasma concentration				CSF concentration			
Added (ng/ml) donepezil	C. V. %	Mean ± SEM	*N*	Added (ng/ml) donepezil	C. V. %	Mean ± SEM	*N*
Inter-day				Inter-day			
6.25	3.23	6.2 ± 0.2	10	6.25	4.76	6.3 ± 0.3	10
12.5	3.25	12.3 ± 0.4	10	12.5	4.84	12.4 ± 0.6	10
25	3.13	25.6 ± 0.8	10	25	3.54	25.4 ± 0.9	10
50	2.23	49.4 ± 1.1	10	50	3.82	49.7 ± 1.9	10
100	1.73	98.3 ± 1.7	10	100	3.29	100.3 ± 3.3	10
Intra-day				Intra-day			
6.25	4.76	6.3 ± 0.3	3	6.25	6.45	6.2 ± 0.4	3
12.5	4.84	12.4 ± 0.6	3	12.5	1.60	12.5 ± 0.2	3
25	4.59	26.1 ± 1.2	3	25	3.50	25.7 ± 0.9	3
50	2.18	50.5 ± 1.1	3	50	2.42	49.5 ± 1.2	3
100	2.02	99.1 ± 2.0	3	100	2.57	101.1 ± 2.6	3

### Accuracy in Plasma and CSF Samples

Donepezil in plasma samples (6.25, 12.5, 25, 50, and 100 ng/ml) was quantified once a day on 10 different days. The accuracy was 98.4 % (6.25 ng/ml), 98.7 % (12.5 ng/ml), 98.9 % (25 ng/ml), 99.3 % (50 ng/ml), and 99.7 % (100 ng/ml), respectively.

Donepezil in CSF samples at the same concentrations was quantified in the same way. The accuracy was 98.1 % (6.25 ng/ml), 98.3 % (12.5 ng/ml), 99.0 % (25 ng/ml), 99.0 % (50 ng/ml), and 99.1 % (100 ng/ml), respectively.

### Limit of Quantification (LOQ) and Limit of Detection (LOD) in Plasma and CSF Samples

LOQ was calculated as the lowest concentration of samples which were measured with a precision of 20 % and a relative error of ±20 %. The LOD was derived from LOQ according to the equation LOD = (3.3 × LOQ)/10.

The donepezil LOQ in plasma samples was 1.56 ng/ml and the LOD was 0.52 ng/ml. The donepezil LOQ in CSF samples was 3.13 ng/ml and the LOD was 1.03 ng/ml.

### Statistical Analysis

The concentrations of donepezil in human plasma and human CSF were calculated from the areas under the peaks using the calibration curve. The calibration curve was calculated by Prism4 statistical software (Graph Pad Software, USA).

Statistical analysis was performed using software IBM^®^ SPSS^®^ Statistics. The mean and SEM (Standard Error of the Mean) were calculated for plasma and CSF concentrations of donepezil. Difference between sampling intervals (12, 24 h) were evaluated via nonparametric Mann–Whitney *U* test, and the differences were considered significant at a significance level 2*α* = 0.05.

## Results

Sixteen patients regularly taking a standard therapeutic dose of donepezil (10 mg per day) were recruited into this clinical study. The patients were randomly divided into two groups depending on the time of plasma and CSF sampling: 12 or 24 h after donepezil administration. Differences in plasma and CSF donepezil concentrations were found between the 12- and 24-h groups, and the concentration changes are summarised in Table 3.

**Table 3:** Donepezil concentrations in human plasma and cerebrospinal fluid and cerebrospinal concentration (% of plasma)

Time interval	Patient no.	Age	MMSE	Donepezil concentration		CSF concentration (% of plasma)
				Plasma (ng/ml)	CSF (ng/ml)	
12 h	1	74	18	47.18	4.20	8.90
	2	84	15	17.29	Under LOD	–
	3	83	23	24.09	2.42	10.04
	4	82	17	69.26	4.29	6.19
	5	80	18	44.71	5.10	11.40
	6	75	11	38.54	5.41	14.03
	7	83	19	13.92	Under LOD	–
	8	62	7	43.10	4.80	11.14
	9	85	22	61.38	10.10	16.45
Mean ± SEM				**39.99** **±** **5.90**	**5.19** **±** **0.83**	
24 h	1	79	22	29.47	6.24	21.02
	2	73	18	26.93	6.65	24.91
	3	79	27	27.16	5.41	19.85
	4	88	24	40.30	8.62	21.34
	5	78	15	27.14	7.42	27.31
	6	71	17	27.34	9.85	35.90
	7	72	20	27.33	8.62	31.50
Mean ± SEM				**29.38** **±** **1.71**	**7.54** **±** **0.55**	

The mean plasma concentration of donepezil was subjectively elevated after 12 h (39.99 ± 5.90 ng/ml) compared with 24-h interval (29.38 ± 1.71 ng/ml), although this difference was not statistically significant (*U* = 22.0, *Z* = −1.006, *p* = 0.35). In contrast, elevated CSF concentration of donepezil was found after 24 h (7.54 ± 0.55 ng/ml) compared with 12-h interval (5.19 ± 0.83 ng/ml; results exclude concentrations under LOD), and this effect was statistically significant (*U* = 7.5, *Z* = −2.177, *p* = 0.03). No correlation between plasma and CSF concentrations of donepezil was found.

## Discussion

The profile of donepezil with respect to its absorption, metabolic pathways, drug–drug interaction, and clearance is well established (Seltzer 2005). Hence, we focused here on the long-term 3-month steady-state concentration profile of donepezil in the plasma and CSF of AD patients, and its fluctuations between 12 and 24 h post dosing. This study contributed to broaden our information on plasma and CSF donepezil concentrations, particularly with respect to donepezil concentration fluctuation after 12 and 24 h post morning dosage which was rare and limited until this time (Tiseo et al. 1998; Darreh-Shori et al. 2006; Jelic and Darreh-Shori 2010). The plasma concentrations of donepezil were 30 ng/ml in patients receiving 5 mg/day and 60 ng/ml in patients receiving 10 mg/day of the drug. Reports pertaining to the distribution of donepezil in the CSF are very scarce. However, Darreh-Shori et al. (2006) showed that the long-term donepezil concentration profile in the CSF differs from that in the plasma. The CSF donepezil concentration appears to be approximately tenfold lower compared with plasma levels but exhibits a similar dose-proportional pattern. In addition, there are indications of the pharmacokinetics of donepezil in the CSF but not in the plasma (Darreh-Shori et al. 2006). The donepezil concentration in the CSF has been found to increase by 50 % between 12 and 24 months of treatment in AD patients receiving the same dose at both intervals (Darreh-shori et al. 2006). This study demonstrated a clear time dependence of the pharmacokinetics of donepezil because the CSF concentration at 24 h after the last administration was approximately one-third higher than that after 12 h. The low concentration of donepezil in plasma and a CSF concentration below LOD in two of the patients were most likely due to noncompliance. The concentration of donepezil in CSF achieved 11.25 ± 1.18 and 25.97 ± 2.09 % 12 and 24 h post administration, compared with actual plasma concentrations (*n* = *7,* mean ± SEM), respectively.

Modern imaging methods showed that the dose of donepezil 5 and 10 mg inhibited cortical AChE activity by only 20–40 % in vivo (Kuhl et al. 2000). These findings suggest that higher doses of donepezil provide greater inhibition and better clinical effects. A dose of 23 mg of donepezil per day was shown to be more effective than lower doses (5 and 10 mg) and was well tolerated (Sabbagh and Cummings 2011). The pharmacological effects depend not only on the dose increase but also on the drug formulation (sustained-release tablets with 23 mg vs. immediate-release tablets with 5 and 10 mg). The higher dosage is recommended by the Expert Working Group (EWG) for the treatment of moderate-to-severe AD (monotherapy or in combination with memantine) (Cummings et al. 2013).

These clinical data support higher dosing of donepezil in the treatment of AD. In the EU, the maximum recommended dose is 10 mg daily, and in the USA the FDA-approved dose is 23 mg daily (Doody et al. 2008; Farlow et al. 2010; Noetzli and Eap 2013).

The other factor that may influence the final donepezil concentration in the CSF of AD patients is the active efflux mechanism across the blood–brain barrier (BBB). According to previously published studies, P-gp (P-glycoprotein) is an important determinant for the disposition of donepezil, influencing its efflux from the central compartment to the blood stream (Ishiwata et al. 2007). It should be noted that BBB P-gp is degraded in the progressive pathogenesis of AD and hence a higher CSF donepezil concentration may be found in patients in an advanced stage of the disease (Hartz et al. 2010).

The limitation of this study is relatively small sample size due to the difficulty of recruiting patients consenting to undergo the invasive lumbar puncture procedure. In addition, the compliance of patients could not be reliably assessed. Also, we expected to find higher concentrations of donepezil in plasma and CSF (based on previously published clinical studies focused on plasma concentration, and also according to our in vivo preclinical data, *Jana Zdarova Karasova personal unpublished result*). On the other hand, donepezil concentration measured by the selected method was well detected also in low concentrations. Despite these limitations, our study is a original work focused on donepezil concentrations in the CSF between doses. Further studies with larger numbers of patients are necessary to verify our results.

## Conclusion

This study supported the use of higher doses of donepezil in the treatment of AD, since its concentration in the CSF is relatively low and unstable throughout the whole day. The CSF concentration of donepezil achieved 25.97 ± 2.09 % of plasma concentration in 24 h and only 11.25 ± 1.18 % in 12 h compared with actual plasma concentrations (*n* = *7,* mean ± SEM). Based on CSF data, it is plausible to predict that donepezil might produce a stronger AChE inhibition in the brain at 24 h compared with 12 h following the administration. This information may help physicians individually adjust the time of drug administration in the patients according to time course of the disease symptoms.

## Abbreviation

AD: Alzheimer’s disease
AChE: Acetylcholinesterase
AUCss: Area under the concentration–time curve at steady state
BBB: Blood–brain barrier
Cap: Capsule
Cmax,ss: Maximum steady-state plasma drug concentration during a dosage interval
CLtot: Total systemic clearance
CSF: Cerebrospinal fluid
CYP: Cytochrome P450
CV: Coefficient of variation
ER: Extended-release formulation
FDA: Food and drug administration
HPLC: High-performance liquid chromatography
IR: Immediate-release formulation
P-gp: P-glycoprotein
SEM: Standard error of mean
SR: Sustained-release formulation
t½: Elimination half-life
tmax,ss: Time to reach Cmax,ss
UGT: Uridine 50-diphosphoglucuronosyltransferase
Vd: Apparent volume of distribution

## References

[CR1] Amini H, Ahmadiani A (2010). High-performance liquid chromatographic determination of rivastigmine in human plasma for application in pharmacokinetic studies. Iran J Pharm Res.

[CR2] Cummings JL, Geldmacher D, Farlow M, Sabbagh M, Christensen D, Betz P (2013). High-dose donepezil (23 mg/day) for the treatment of moderate and severe Alzheimer’s disease: drug profile and clinical guidelines. CNS Neurosci Ther.

[CR3] Darreh-Shori T, Meurling L, Pettersson T, Hugosson K, Hellström-Lindahl E, Andreasen N, Minthon L, Nordberg A (2006). Changes in the activity and protein levels of CSF acetylcholinesterases in relation to cognitive function of patients with mild Alzheimer’s disease following chronic donepezil treatment. J Neural Transm.

[CR4] Doody RS, Corey-Bloom J, Zhang R, Li H, Ieni J, Schindler R (2008). Safety and tolerability of donepezil at doses up to 20 mg/day: results from a pilot study in patients with Alzheimer’s disease. Drugs Aging.

[CR5] Farlow MR, Salloway S, Tariot PN, Yardley J, Moline ML, Wang Q, Brand-Schieber E, Zou H, Hsu T, Satlin A (2010). Effectiveness and tolerability of high-dose (23 mg/d) versus standard-dose (10 mg/d) donepezil in moderate to severe Alzheimer’s disease: a 24-week, randomized, double-blind study. Clin Ther.

[CR6] Giacobini E (2008). Cholinergic foundations of Alzheimer’s disease therapy. J Physiol Paris.

[CR7] Hartz AMS, Miller DS, Bauer B (2010). Restoring blood-brain barrier P-glycoprotein reduces brain amyloid in a mouse model of Alzheimer’s disease. Mol Pharmacol.

[CR8] Hort J, O´Brien JT, Gainotti G G, Pirttila T, Popescu BO, Rektorova I, Sorbi S, Scheltens P (2010). EFNS guidelines for the diagnosis and management of Alzheimer’s disease. Eur J Neurol.

[CR9] Ishiwata K, Kawamura K, Yanai K, Hendrikse NH (2007). In vivo evaluation of P-glycoprotein modulation of 8 PET radioligands used clinically. J Nucl Med.

[CR10] Jelic V, Darreh–Shori T (2010). Donepezil: a review of pharmacological characteristics and role in the management of alzheimer disease. Clin Med Insights Ther.

[CR11] Kuhl DE, Minoshima S, Frey KA, Foster NL, Kilbourn MR, Koeppe RA (2000). Limited donepezil inhibition of acetylcholinesterase measured with positron emission tomography in living Alzheimer cerebral cortex. Ann Neurol.

[CR12] McKhann G, Drachman D, Folstein M, Katzman R, Price D, Stadlan EM (1984). Clinical diagnosis of Alzheimer’s disease: report of the NINCDS-ADRDA work group under the auspices of Department of Health and Human Services Task Force on Alzheimer’s disease. Neurology.

[CR13] Noetzli M, Eap CB (2013). Pharmacodynamic, pharmacokinetic and pharmacogenetic aspects of drugs used in the treatment of Alzheimer’s disease. Clin Pharmacokinet.

[CR14] Ressner P, Hort J, Rektorova I, Bartos A, Rusina R, Linek V, Sheardova K (2008). Recommendations for the diagnosis and management of Alzheimer´s disease and other disorders associated with dementia. Cesk Slov Neurol N.

[CR15] Ritchie CW, Ames D, Clayton T, Rosalind L (2004). Metaanalysis of randomized trials of the efficacy and safety of donepezil, galantamine and rivastigmine for the treatment of Alzheimer disease. Am J Geriat Psychiat.

[CR16] Sabbagh M, Cummings J (2011). Progressive cholinergic decline in Alzheimer’s disease: consideration for treatment with donepezil 23 mg in patients with moderate to severe symptomatology. BMC Neurol.

[CR17] Seltzer B (2005). Donepezil: a review. Expert Opin Drug Metab Toxicol.

[CR18] Talesa VN (2001). Acetylcholinesterase in Alzheimer’s disease. Mech Ageing Dev.

[CR19] Tiseo PJ, Rogers SL, Friedhoff LT (1998). Pharmacokinetic and pharmacodynamic profile of donepezil HCl following evening administration. Br J Clin Pharmacol.

